# Self-Prescribed Analgesic Use for Acute Dental Pain Among Saudi Adults: A Cross-Sectional Study

**DOI:** 10.7759/cureus.102247

**Published:** 2026-01-25

**Authors:** May Alsnani

**Affiliations:** 1 Oral Medicine, Faculty of Dentistry, King Saud University, Riyadh, SAU

**Keywords:** analgesics, dental pain, self-medication, self-prescribed pain relief, toothache

## Abstract

Introduction: Self-medication with analgesics is a common practice for managing dental pain and may delay appropriate dental care. This study aimed to assess the prevalence, awareness, and practices related to self-prescribed analgesic use for toothache among adults living in Saudi Arabia.

Methods: A cross-sectional survey was conducted over one week using an online convenience sampling method targeting adults aged ≥18 years across five regions of Saudi Arabia. Data were collected using a structured Arabic questionnaire covering sociodemographic characteristics, dental history, pain characteristics, and self-medication practices. Ethical approval was obtained prior to data collection. Statistical analysis was performed using the Statistical Package for the Social Sciences (SPSS).

Results: A total of 1,588 participants were included, of whom 750 (47.2%) reported using analgesics to manage dental pain. Ibuprofen was the most commonly used analgesic (58%). Alternative pain management methods included herbal remedies (10.8%), rinsing with salted water (5.4%), and seeking medical consultation (4.7%). Multiple pain relief methods were reported by 27.3% of participants. Self-prescribed analgesic use was significantly associated with age (p = 0.005), marital status (p = 0.017), region of residence (p < 0.0001), dental insurance status (p = 0.032), pain source (p = 0.033), and pain severity (p < 0.0001).

Conclusions: Self-medication with analgesics for dental pain is highly prevalent among adults in Saudi Arabia and is influenced by demographic and pain-related factors. These findings highlight the need for public health interventions focusing on patient education regarding the risks of self-medication and improving access to timely and affordable dental care.

## Introduction

Analgesics are a group of medications used for the management of pain [1]. However, identifying and treating the underlying cause of pain is essential for effective relief. Individuals with dental fear often resort to over-the-counter (OTC) analgesics to avoid dental visits [2]. Although OTC medications are generally safe when used at therapeutic doses according to guidelines, adverse effects can occur when they are misused [3].

According to the World Health Organization, self-medication is defined as the selection and use of medicines by individuals to treat self-recognized symptoms or disorders [4]. This practice is not limited to oral health conditions but also extends to systemic issues, such as fever, cough, and headache. The tendency toward self-prescription is multifactorial, influenced by factors such as time constraints, reusing prescriptions, socioeconomic status, inaccessibility to qualified dental care, and dental anxiety or phobia [5].

Acute dental pain is defined as a sudden-onset, short-term pain originating from the teeth or surrounding oral structures, typically caused by conditions such as dental caries, pulpitis, periodontal inflammation, dental trauma, or acute infection. It is usually characterized as severe and persistent, often leading individuals to consume large quantities of OTC analgesics for relief. Moreover, the systematic review by Moore et al. concluded that the use of ibuprofen with paracetamol is the most commonly used OTC as a single dose for acute post-operative pain [6,7]. However, misuse of paracetamol in the management of dental pain has led to cases of overdose, which can result in irreversible liver damage and even death [8]. The symptoms of overdose vary depending on the time since ingestion, and because many of these symptoms are nonspecific, obtaining a detailed drug history is essential when patients present to emergency departments with dental pain [8].

Previous studies in Saudi Arabia have reported a high prevalence of self-medication with analgesics; however, most have focused on specific subpopulations, such as health sciences students, rather than the general adult population. There remains a lack of comprehensive, community-based data on self-prescribed analgesic use specifically for dental pain across different regions of the country.

Therefore, this study aimed to estimate the prevalence and describe the patterns of self‑prescribed analgesic use for toothache among adults living in Saudi Arabia. Secondary objectives were to examine the associations between this practice and key sociodemographic, economic, and behavioral factors, with the goal of generating evidence to inform targeted public health initiatives and policy planning, without inferring causality.

## Materials and methods

Study design

This study employed a cross-sectional survey design to assess the prevalence and determinants of self-prescribed analgesic use for acute dental pain among adults in Saudi Arabia.

Study setting

We recruited participants from the five main regions of Saudi Arabia: Central (Riyadh, Qassim), Eastern (Dammam, Al-Ahsa), Western (Jeddah, Mecca), Northern (Hail, Tabuk), and Southern (Abha, Jazan). We selected the study areas to ensure geographical diversity across the country. These regions represent Saudi Arabia's primary administrative divisions, ensuring coverage of urban, semiurban, and rural populations. Regional stratification helps capture socioeconomic, cultural, and healthcare access variations that may influence self-medication practices. This study was conducted over one week using an online convenience sampling method. The minimum sample size was. The minimum sample size was calculated using a prevalence estimate of 68.6% for self-medication with analgesics, as reported in a previous study conducted among adults in Saudi Arabia [9], a 95% confidence interval, and a 5% margin of error, resulting in a sample size of at least 1,500 participants. The survey link was distributed electronically via social media platforms and community networks to maximize reach across different regions. Ultimately, 1,588 individuals participated in the study.

Inclusion criteria

The study included adults aged 18 years and above, residents of Saudi Arabia at the time of data collection, individuals who had experienced dental pain within the past 12 months, individuals who were able to read and understand the survey (in Arabic), and those who provided informed consent to participate in the study.

Exclusion criteria

The study excluded individuals below 18 years of age, nonresidents or temporary visitors to Saudi Arabia, participants who had not experienced any form of dental pain in the past year, individuals currently under professional dental treatment for chronic conditions (e.g., ongoing prescribed pain management), and those who did not complete or provided inconsistent responses in the survey questionnaire.

Study measures

We developed a structured, closed-ended questionnaire in Arabic to collect data (see the Appendix). The questionnaire consisted of three main sections: (1) demographic characteristics: age, gender, marital status, region of residence, education level, and dental insurance status; (2) dental-related questions: frequency of dental visits, history of toothache, source and severity of pain (measured by a numerical pain scale), and previous dental treatments; (3) self-medication practices: types of analgesics used, frequency and dosage, reasons for self-medication, and other home remedies (e.g., herbs and salted water). The questionnaire was pretested on a small sample (n = 30) to ensure clarity and reliability, with necessary modifications made before its full deployment.

Data collection

The survey was administered online using Google Forms (Google LLC, Mountain View, CA) over one week. Participation was voluntary, and respondents could withdraw at any stage.

Ethical statement

The study protocol was reviewed and approved by the Institutional Review Board (IRB)/Ethical Committee of the Dental College at King Saud University (IRB Approval No. E-20-4572) and performed in accordance with the Declaration of Helsinki. Informed consent was obtained electronically from all participants before commencing the survey. All data were collected anonymously and stored securely to ensure confidentiality.

Statistical analysis

Data were analyzed using Statistical Product and Service Solutions (SPSS, version 21.0; IBM SPSS Statistics for Windows, Armonk, NY). Descriptive statistics (frequencies, percentages, means, and standard deviations) were used to summarize participant characteristics and self-medication practices. Inferential statistics, including Pearson's chi-square test and Student's t-test for independent samples, were applied to assess associations

## Results

A total of 1,588 adults from across Saudi Arabia participated in the study. Of these, 772 participants (49.6%) were aged over 40 years, and 1,336 (84.1%) were women. The vast majority were Saudi nationals, with 1,553 individuals (97.8%) being identified as Saudi. Most participants were married, accounting for 1,219 respondents (76.8%). Regarding socioeconomic status, 763 participants (48.0%) reported a monthly income greater than 10,000 Saudi Riyals. The central region was the most represented, with 994 participants (62.6%) residing there.

Prevalence and methods of toothache management

Out of all participants, 750 individuals (47.2%) reported using analgesics to manage a toothache. Herbal remedies were used by 172 participants (10.8%), while 86 (5.4%) relied on rinsing with salted water, and 75 (4.7%) visited a doctor for relief. Notably, 433 participants (27.3%) reported using more than one method to alleviate their dental pain (Table 1).

**Table 1:** Prevalence of the methods used to relieve dental pain. Over-the-counter painkillers refer to non-prescription analgesic medications used by participants for dental pain relief. Specific types of analgesics are illustrated separately in Figure 3.

Type of items	n (%)
Over-the-counter painkiller	750 (47)
Salted water	86 (5)
Ice packs	6 (0.4)
Herbs	172 (11)
Nothing	64 (4)
Visit the doctor	75 (5)
Two or more items	435 (27)

Sources of information and barriers to dental care

**Figure 1:**
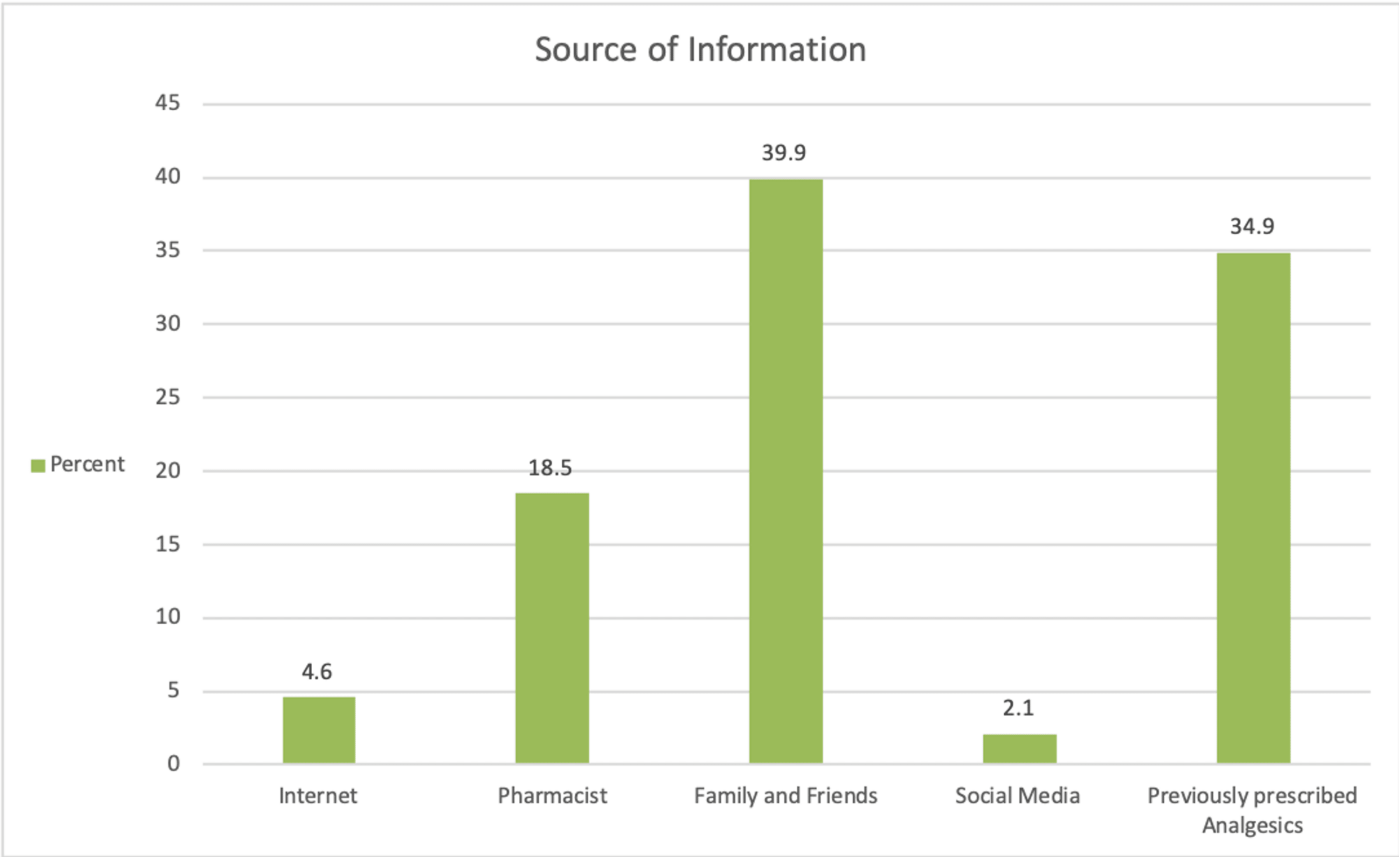
Source of information and recommendation.

**Figure 2:**
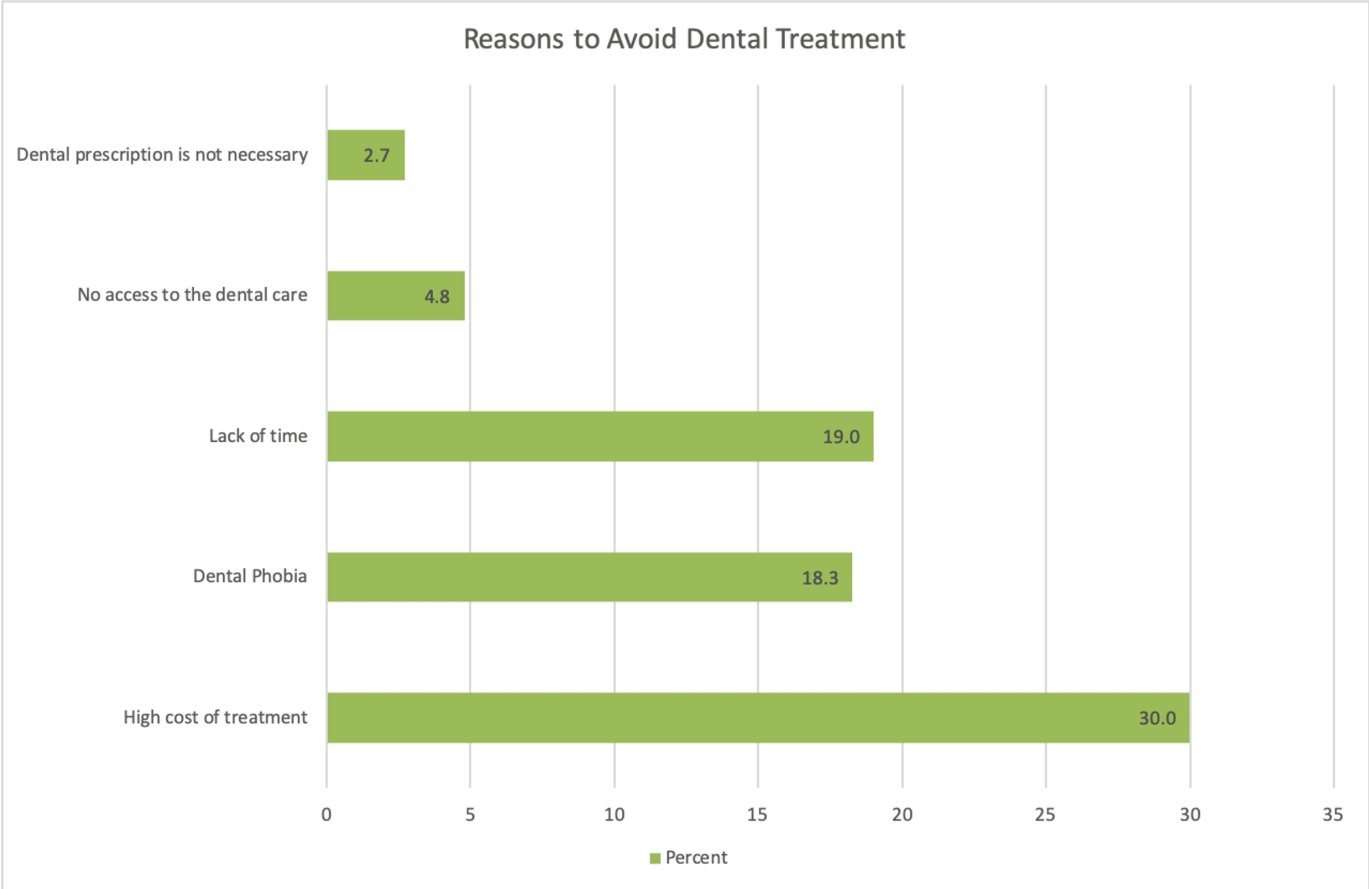
Reasons to avoid dental visit in case of toothache.

When asked about their main source of information or recommendations for managing a toothache, 635 participants (40.0%) indicated family and friends. The most commonly reported barrier to seeking professional dental care was the high cost of treatment, expressed by 476 respondents (30.0%). Other reported barriers included lack of time, fear of dental procedures, and the perception that the pain was not severe enough to require a dental visit (Figures 1, 2).

Preferences for analgesics

**Figure 3:**
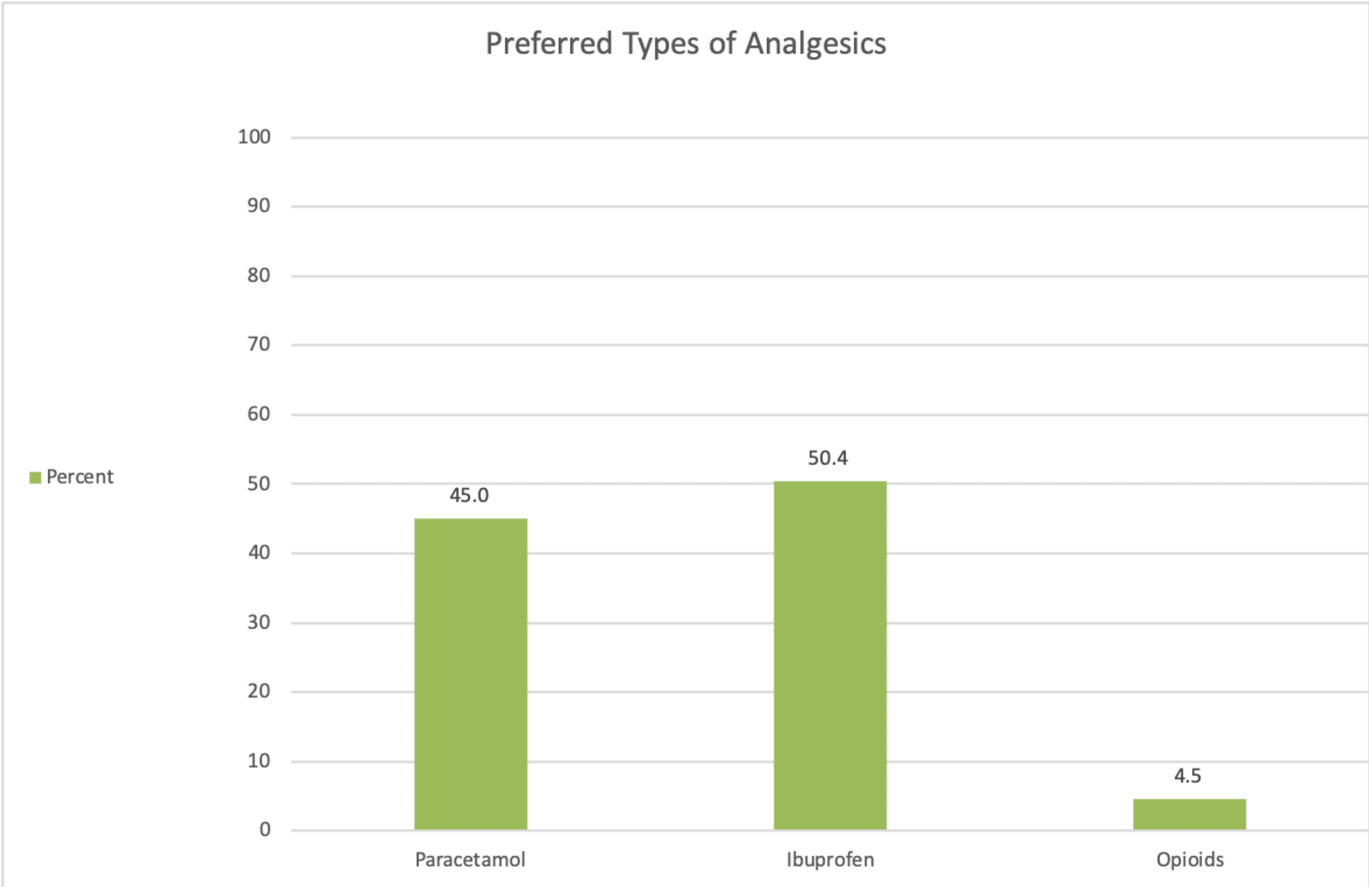
Preferred types of analgesics used for acute dental pain. The figure illustrates the distribution of specific analgesic classes used by participants who reported over-the-counter painkiller use, including paracetamol, ibuprofen, and opioids.

Among those who used analgesics, ibuprofen was the most frequently chosen medication, preferred by 435 participants (58%). Other analgesics, such as paracetamol, were selected less often (Figure 3).

Associations with sociodemographic and clinical factors

The statistical analysis demonstrated that several sociodemographic and clinical characteristics were significantly associated with the use of analgesics for toothache. Participants aged over 40 years showed a higher proportion of analgesic use (p = 0.005). Marital status also played a role, as married individuals were more likely to use analgesics compared to single participants (p = 0.017). Regional differences were evident, with participants from the central region reporting the highest prevalence of analgesic use (p < 0.0001) (Table 2).

**Table 2:** Association between the sociodemographic characteristics and the use of nonprescription analgesics to relieve dental pain. *Statistically significant

Characteristics	Analgesics Use, No. (%)		Χ^2^-value	p-value
	Yes (n=941)	No (n=647)		
Age groups			15	0.005*
18-24	120 (51)	117 (49)		
25-29	94 (69)	43 (31)		
30-34	112 (62)	68 (38)		
35-40	146 (56)	116 (44)		
>40	469 (61)	303 (39)		
Gender			0.78	0.37
Male	143 (57)	109 (43)		
Female	798 (60)	538 (40)		
Nationality			0.6	0.4
Saudi	918 (59)	635 (41)		
Non-Saudi	23 (66)	12 (34)		
Marital Status			5.7	0.02*
Single	199 (54)	170 (46)		
Married	742 (61)	477 (39)		
Educational Status			0.8	0.9
Uneducated	4 (67)	2 (33)		
High School or less	226 (58)	167 (43)		
Bachelor’s degree	626 (60)	422 (40)		
Higher studies	85 (60)	56 (40)		
Economic Status (in SR)			2.4	0.3
3000-5000	191 (60)	126 (40)		
6000-9000	287 (57)	221 (44)		
>10,000	463 (61)	300 (39)		
Region			25	< 0.0001*
Southern	95 (57)	71 (43)		
Eastern	122 (49)	128 (51)		
Northern	22 (50)	22 (50)		
Western	98 (73)	36 (27)		
Central	604 (61)	390 (39)		

The lack of dental insurance significantly increased the use of analgesics for dental pain (p = 0.032). The specific source of pain from the tooth itself was also associated with their likelihood of using analgesics (p = 0.033). Furthermore, participants who reported higher pain scores were significantly more likely to use analgesics for relief (p < 0.0001) (Table 3).

**Table 3:** Association between dental care services and the use of nonprescription analgesics to relieve dental pain. *Statistically significant, SD: standard deviation

Variables	Analgesics Use, No. (%)		Χ^2^-value/t-value	p-value
	Yes (n=941)	No (n=647)		
Do you treat dental problems in government sector?			0.9	0.4
Yes	218 (57)	163 (43)		
No	723 (60)	484 (40)		
Do you have dental Insurance?			4.6	0.03*
Yes	248 (64)	140 (36)		
No	693 (58)	507 (42)		
How often visit dentist?			3.3	0.1
Only in emergency	761 (60)	499 (40)		
Regularly (6 to 12 months)	180 (55)	148 (45)		
Experienced toothache?			1.3	0.3
Yes	930 (59)	635 (41)		
No	11 (48)	12 (52)		
Source of tooth pain?			8.7	0.03*
The tooth	523 (62.5)	314 (37.5)		
The gums	16 (50)	16 (50)		
Both	391(56.2)	305(43.8)		
Other	11(47.8)	12(52.2)		
Pain score (mean ± sd )	7.24±2.1	6.83±2.2	3.6	<0.0001*

## Discussion

This study provides novel insights by examining the prevalence and patterns of self-prescribed analgesic use for toothache among a large and diverse adult population across all five regions of Saudi Arabia. The primary objective was to estimate the frequency of this practice and explore factors associated with its occurrence, thereby generating evidence relevant to public health planning and dental pain management policies.

The prevalence of self-prescribed analgesic use for toothache was high, with 59.2% of respondents reporting this behavior. Despite strict national regulations limiting non-prescription medication dispensing [10], the persistence of self-medication highlights ongoing challenges related to health literacy, access to care, and patient education. These findings are consistent with previous Saudi studies reporting prevalence rates between 50.4% and 81.4% [11,12], underscoring the continued relevance of this issue for national oral health strategies.

Several demographic and socioeconomic factors were significantly associated with analgesic use for toothache, including age, marital status, and geographic region (p < 0.05). While earlier Saudi studies have documented variations by age and sex, this study adds new evidence regarding regional differences [12]. These disparities may reflect unequal access to dental services, particularly in less urbanized areas, where barriers such as limited clinic availability, travel distance, and reduced awareness of services are more pronounced. This finding highlights the importance of region-specific interventions to improve equitable access to dental care.

Economic factors were also significantly associated with self-prescribed analgesic use. Participants reporting higher dental costs or lack of insurance were more likely to rely on analgesics (p = 0.032), consistent with international evidence linking out-of-pocket healthcare expenditures to self-medication behaviors [13-15]. Similar trends have been reported in Saudi Arabia, where limited insurance coverage and difficulty accessing dental services contribute to self-management of dental pain [11]. These findings are particularly relevant within the context of Saudi Vision 2030, which aims to reduce financial barriers to healthcare access.

Pain intensity showed a strong association with analgesic use (p < 0.0001), supporting previous evidence that severe dental pain is a primary trigger for self-medication [12,16]. Ibuprofen was the most commonly used analgesic (58%), reflecting widespread reliance on nonsteroidal anti-inflammatory drugs (NSAIDs) for dental pain management locally and internationally [11,16-18]. Although NSAIDs are generally perceived as effective and safe for short-term use, their potential adverse effects, including gastrointestinal and renal complications, emphasize the need for improved public awareness regarding appropriate medication use. Notably, only 4.7% of participants reported seeking professional medical care for a toothache, while more than one-quarter used multiple self-management methods. Such practices may delay definitive dental treatment and obscure underlying pathology, consistent with findings from prior self-medication research [19].

Overall, the high prevalence of self-medication with analgesics for toothache management in Saudi Arabia underscores the urgent need for public health intervention. Although this phenomenon is not unique to Saudi Arabia, similar trends have been observed in countries with limited healthcare accessibility. Targeted measures should focus on improving access to affordable dental care, increasing public awareness about the risks of self-medication, and promoting the proper use of over-the-counter medications. Furthermore, pharmacists play a pivotal role in patient education and should be empowered to provide counseling on the safe and appropriate use of analgesics, given that pharmacies remain the primary source of these medications [11,20].

Strengths and limitations

This study has several strengths. The large sample size (n = 1,588) and inclusion of participants from all five regions of Saudi Arabia enhance the breadth of perspectives captured and support cautious generalization to the adult population at a national level. The use of a structured questionnaire allowed for a comprehensive assessment of demographic, socioeconomic, and pain-related factors associated with self-prescribed analgesic use for toothache. In addition, ethical approval and standardized data collection procedures contributed to methodological transparency.

Nevertheless, several limitations should be acknowledged. The cross-sectional design precludes causal inference, and the use of convenience sampling and online data collection may have introduced selection bias, as certain population groups may have been overrepresented. Although participants were recruited from all major regions, not all cities within each region were represented, which may limit the geographic diversity of the sample. The reliance on self-reported data also introduces the possibility of recall bias and social desirability bias, potentially affecting the accuracy of reported behaviors.

Furthermore, the questionnaire used in this study was not formally validated, which may influence the reliability and precision of the measured variables. The analysis was limited to bivariate statistical methods, and the absence of multivariable modeling restricts the ability to control for potential confounding factors. In addition, clinical outcomes and adverse effects related to self-prescribed analgesic use were not assessed, which limits insight into the health consequences of these practices.

These methodological constraints may have influenced prevalence estimates and the observed associations. Future studies should consider probability-based sampling, inclusion of a broader range of cities, formal validation of survey instruments, multivariable analytical approaches, and the incorporation of objective clinical assessments to strengthen evidence and further inform public health strategies.

## Conclusions

This study highlights the widespread practice of self-prescribed analgesic use for toothache among adults in Saudi Arabia and identifies critical sociodemographic, economic, and behavioral factors associated with this behavior. The findings point to persistent gaps in dental care access and awareness, as well as a preference for over-the-counter pain management over professional dental consultation. These patterns suggest a risk of delay in addressing underlying dental conditions and a heightened potential for misuse of analgesics.

Given these insights, targeted public health interventions and education campaigns are warranted to promote appropriate pain management and encourage timely dental care-seeking. Additionally, more comprehensive insurance coverage, enhanced pharmacist engagement, and equitable healthcare distribution may be key strategies to address this public health concern. Future research should build on these findings to guide effective policy and clinical practice.

## Appendices

Demographic data

Below presents the demographic data for the study.

Gender: (Male, Female)

Nationality: (Saudi-Saudi)

Age: (19-24, 25-29, 30-34, 35-40, Above 40)

Region: (Middle region, Eastern region, Western region, Northern region, Southern region)

Marital status: (Single, Married)

Socioeconomic status: Low (3000-5000 SR), Middle (6000-9000 SR), High (10,000 SR or above)

Level of education: Non-educated, High school or less, Bachelor's degree, Higher studies

Do you treat dental problems in the government sector?: Yes, No

Do you have dental insurance?: Covered, Not covered

General Dental-Related Questions

How often do you visit a dentist?: Emergency only, Regularly every 6-12 months

Have you or any of your close relatives ever had a toothache?: Yes, No

If your answer is yes, what actions do you take to relieve the pain?: Over-the-counter painkiller, Salted water, Ice packs, Herbs (myrrh, clove, etc.), Nothing

Pain Description Questions

The source of pain you or your relatives have experienced most of the time is: The tooth, The gums, Both, Others

Duration of the pain: Hours/less than a day, 1-2 days, More than a week

How do you describe the pain?: Mild, Moderate, Severe

Analgesics-Related Questions

What type of analgesics did you use to relieve pain in case of a toothache?: Paracetamol, Ibuprofen, Opioids, Others

What's the duration of the analgesic intake? 1 day, 2-3 days, Week or more

How long does the analgesic you previously used to relieve your pain take to start working?: Immediately, Minutes, Hours

What is the source of the information or advice taken from whom?: Google or the internet, Pharmacist, Family and friends, Previously prescribed analgesics

Reasons for avoiding a dental visit in such a case:  High-cost treatment, Dental phobia, Lack of time, No access to dental care, Dental prescription is not necessary

Are you aware of the risks or hazards of the drug you used to relieve your tooth pain? No hazards, Overdose, Addiction, Adverse drug reactions, Damaging body organs, Worsening the existing condition
